# Comparative screening of single nucleotide polymorphisms in β-casein and κ-casein gene in different livestock breeds of India

**DOI:** 10.1016/j.mgene.2015.03.005

**Published:** 2015-04-13

**Authors:** Lakshya Veer Singh, S. Jayakumar, Anurodh Sharma, Shishir Kumar Gupta, S.P. Dixit, Neelam Gupta, S.C. Gupta

**Affiliations:** a DNA Fingerprinting Unit, National Bureau of Animal Genetic Resources, Karnal 132001, India; b Krishi Bhawan, ICAR, New Delhi, India; c Division of Veterinary Biotechnology, Indian Veterinary Research Institute, Izatnagar, Bareilly 243122, India

**Keywords:** β-casein gene, κ-casein, SNPs, Livestock breeds

## Abstract

The most polymorphic milk protein gene is β-casein; 13 protein variants are known in cattle. Milk protein genetic polymorphism has received considerable research interest in recent years because of possible associations between milk protein and economically important traits in livestock. The present study was undertaken to explore the genetic polymorphisms in exon 7 of β-casein and exon 4 of κ-casein genes in Arunachali yaks (*Bos grunniens*), Sahiwal (*Bos indicus*) cattle, malpura sheep (*Ovis aries*) and Gaddi goat (*Capra hircus*). Results of the study revealed presence of 11 SNP variants in all livestock species. Four SNPs were observed in *Bos indicus*; two SNPs in *Bos grunniens*; three SNPs in *Ovis aries* and three SNPs in *Capra hircus*. These variations are found to be synonymous in nature as these variations do not result in their corresponding amino acids. A total of five polymorphic sites have been described at the κ-casein (CSN3) locus in the Indian domestic Gaddi goat (*Capra hircus*) when compared with exotic goat (X60763) while sequence analysis of κ-casein gene in sheep showed three novel nucleotide changes in malpura sheep when compared with the exotic sheep (AY237637). These results highlight the importance of taking into consideration the CSN3 SNPs when performing selection for milk composition in dairy livestock breeds.

## Highlights

• This is first comparative sequence analysis of Beta casein and kappa casein genes in different livestock breeds of India.
• Total 11 SNPs variants were found in all livestock species studied, namely, *Bos indicus*, *Bos grunniens*, *Ovis aries* and *Capra hircus*.
• Four SNPs were observed in *Bos indicus*; two SNPs in *Bos grunniens*; three SNPs in *Ovis aries* and three SNPs in *Capra hircus*.
• Homology of 99% with *Bos taurus* and *Bos indicus*, 97% with *Bubalus bubaline*, *99*% with *Bos grunniens*, 95% with *Ovis aries*, 95% with *Capra hircus*.
• Sequence analysis of κ-casein gene in sheep showed three novel nucleotide changes in malpura sheep when compared with the exotic sheep (AY237637).

## Introduction

Finding effective genetic markers for economically important traits in farm animals, especially those which allow for faster and more precise prediction of breeding values, has been a challenge in animal genetics for decades. Bovine chromosome 6 is an object of remarkable interest of many research groups searching for QTL for milk production traits.

More than 95% of the proteins contained in ruminants' milk (mainly goats, sheep and cattle) are synthesized from 6 structural genes encoding proteins: α-lactalbumin and β-lactoglobulin, the two main whey proteins in ruminants, and the four caseins αs1, αs2, β and K which are encoded by four tightly linked and clustered genes (Ferretti et al., 1990, Threadgill and Womack, 1990). β-casein is a member of casein cluster and with 13 protein variants known; it is the most polymorphic milk protein gene.

Four casein genes (CSN1S1, CSN2, CSN1S2 and CSN3, respectively) are located as a cluster on chromosome 6 in cattle, sheep, and goats (Ferretti et al., 1990, Hayes et al., 1993, Popescu et al., 1996). The discovery of milk protein polymorphism (Aschaffenburg and Drewry, 1955) and its impact on milk production and technological properties has promoted characterization of milk protein variants in different species Grosclaude et al. (1987); (Moioli et al., 1998). Polymorphism of casein genes have been well studied in cattle and goat (Caroli et al., 2009, Marletta et al., 2007, Rando et al., 2000) and the variants have been found to be associated with the composition and biological properties of milk (Martin et al., 2002, Vinesh et al., 2013).

The present study is focusing on the genetic polymorphism of casein protein genes, which are strongly related to economically important milk quantitative traits in Sahiwal cattle, Arunachali yak, malpura sheep and Gaddi goat breeds, as a tool for genetic improvement of these breeds. On the other hand, promoting selection programs that are depending on the use of genetic markers will help in improving breeding strategies. This study aimed to identify the genetic polymorphism of β-casein and κ-casein genes in Sahiwal cattle, Arunachali yak, malpura sheep and Gaddi goat.

## Materials and methods

The present study was conducted in a panel of 48 animals belonging to different species; viz. Sahiwal cattle (*N* = 12), Arunachali yak (*N* = 12), malpura sheep (*N* = 12) and Gaddi goat (*N* = 12). Approximately 8–10 ml blood was collected from each animal in EDTA coated vacutainer tubes and stored in deep freezer at − 20 °C until the isolation of DNA. Genomic DNA was isolated using the phenol-chloroform method (Sambrook et al., 1989). The quality and quantity of isolated DNA were determined using agarose gel electrophoresis (0.8%) and a Nanodrop spectrophotometer (GE Healthcare). Purified DNA ran as a single band on agarose gel and the OD 260 > 280 ratio for all the samples was between 1.8 indicating good quality of extracted DNA.

### PCR amplification

The exon 7 (467 bp) of β-casein and exon 4 of κ-casein gene were successfully amplified using the primers reported by Bonifacio et al. (2001) in Arunachali yaks, Sahiwal cattle, Malpura sheep and Gaddi goat (F 5′CTTCTTTCCAGGATGAAGTCC-3′, R 5′GACTTACAAGAATAGGGAAGG3′) and exon 4 (κ-CSNF-5′GAGAAAGATGAAAGATTCTTC-3′ and primer κ-CSN R-5′-GCTTCTGGATTATCTACAGTG-3′) Custom DNA sequencing has been done to find out polymorphisms in the above livestock breeds. PCR amplification was performed in a total volume of 25 μl containing ~ 50–100 ng of genomic DNA, 10 pmol of each primer, 200 μM of each dNTP, 2.5 μl of 10 × buffer with 1.5 mM MgCl_2_ and 1 U Taq DNA polymerase (Bangalore Genei Pvt. Ltd., Bangalore, India). PCR amplification was performed using Eppendorf (Germany) with thermal cycling conditions of initial denaturation at 95 °C for 5 min followed by 30 cycles of 94 °C for 30 s, annealing temperature (60 °C) for 30 s and 72 °C for 30 s followed by final extension at 72 °C for 5 min. An amplified product was analyzed by electrophoresis on 1.5% agarose gel at 100 V for 20 min using ethidium bromide staining.

### PCR cleanup and DNA sequencing

Amplified PCR products were purified by an enzymatic method using exonuclease 1 and Antarctic phosphatase. Purified PCR products were sequenced with both the forward and reverse primers using a BigDye Terminator cycle sequencing kit (Applied Biosystems, USA) on an automated Genetic Analyzer ABI 3100 (Applied Biosystems, USA).

### Sequence analysis

The Sequence data were edited manually using Chromas Ver.2.33, (http >www.technelysium.com au > chromas.html). Multiple sequence alignments were performed using MegAlign tool of LASERGENE software (DNA STAR, Inc., Madison, WI, USA). Genotype frequencies of single nucleotide polymorphism (SNPs) were determined by direct counting method. The coding DNA sequence of exon 7 of β-casein and exon 4 of κ-casein genes were conceptually translated to amino acid sequence using EDIT Seq tool of DNASTAR software. Nucleotide BLAST program at NCBI (http:www.ncbi.nlm.nih.gov.BLAST) was used for sequence homology searches in public databases.

## Results and discussion

Genomic selection is the state of the art today and MAS could be implemented in the genomic selection. The studies of genetic marker applied to animal breeding and production are focused mainly on analyses of mutations located within candidate genes of quantitative traits (Singh et al., 2014). Because of the economic importance of the livestock industry, it appears clearly essential to reveal some quality information related to casein gene. In this study, variations and gene specific polymorphisms in the various livestock species of β-casein gene were investigated based on automated DNA sequencing and SNP detection, which allowed the detection of novel allele as well as status of previously reported alleles. SNP analysis is a well-established tool for the identification of genes associated with traits of economic interest in livestock populations (He et al., 2009, Lai et al., 2009, Singh et al., 2011).

Collectively, 467 bp fragment of β-casein gene was sequenced and compared to the equivalent reference. Overall 11 single nucleotide polymorphisms (SNPs) were identified, resulting in an average density of 1 SNP for every 42 bp sequence. Out of 11 SNPs identified, 72% (*N* = 8) was transitions and 28% (*N* = 3) was transversion. Sequence analysis of the 12 non-related Sahiwal cattle and Arunachali yak animals revealed a total of five SNPs across the exon 7 of the β-casein gene sequence analyzed. Overall 90% (*N* = 5) of the SNPs encountered were transitions and 10% (*N* = 1) were transversion changes in this gene. The sequence information obtained by directing sequencing of the PCR products was used to detect SNPs in the exon 7 of the β-casein gene in the Sahiwal cattle. A fragment of 465 bp of identified four nucleotide variations in exon 7 at the position *g*.8093 A > G, *g*.8101 A > C, *g*.8261 T > C (Heterozygous) and *g*.8461C > T (Heterozygous) in Sahiwal cattle. All the SNPs presented in Table 1.These variations are found to be synonymous in nature as these variations do not result in their corresponding amino acids. The average density of polymorphisms in bovine β-casein gene was 1 SNP per 117 bp sequenced. Casein genetic polymorphisms are important and well known due to their effects on quantitative traits and technological properties of milk (Ceriotti et al., 2004).

A fragment of 467 bp sequence analysis of the amplified samples of the β-casein gene in Arunachali yak revealed two nucleotide substitutions at the position *g*.8261 T > C (transition) and *g*.8412C > T (transition) in exon 7 (Table 1). The average density of polymorphisms in bovine β-casein gene was 1 SNP per 232 bp sequenced. BTA6 is known as containing several QTL with large effects on milk production traits. So far only casein loci were indicated as candidate genes responsible for significant portion of trait variation. CSN2 as a member of casein cluster consisting of genes was indicated as a gene influencing milk performance traits. Polymorphism occurring within exon VII giving rise to A1 and A2 alleles was reported to increase both protein yield and content and simultaneously to decrease the content and yield of milk fat (Velmala et al., 1995, Ikonen et al., 1999, Ikonen et al., 2001, Nilsen et al., 2009, Oleński et al., 2012, Chessa et al., 2003). Sequence data from these samples were entered in the NCBI GenBank database under the following accession numbers: HQ902899 and KF430646.

The sequence analysis revealed three possible substitution mutations in malpura sheep. A fragment of 467 bp corresponding to malpura sheep β-casein gene was investigated. A total of three SNPs were observed when compared with a template sequence (Acc. No. X79703). The average density of polymorphisms in ovine β-casein gene was 1 SNP per 157 bp sequenced. All these SNPs are found to be synonymous in nature as these variations do not result in their corresponding amino acids. All SNPs are given in Table 2. Chessa et al. (2003) reported that new patterns revealed 2 new variants at CSN2 gene. Frequencies of the 2 variants in the samples analyzed were 0.18 and 0.02. The less common variant is characterized by a silent mutation in the triplet coding for Gln192, whereas in the more frequent one a C to A transversion is responsible for the amino acid exchange Leu196 > Ile196. The effects of casein genetic polymorphisms are important due to their impact on quantitative traits and technological properties of milk (Ceriotti et al., 2004). So, caseins have been proposed as polymorphic markers for the selection in order to improve yield and quality of cheese (Bonifacio et al., 2001). Othman et al. (2013) reported that two different patterns showed two single nucleotide substitutions; A > C and C > T without any amino acid exchange. The frequencies of these two different patterns were 96.67% and 3.33% in Rahmani; 65.52% and 34.48% in Ossimi and 88.46% and 11.54% in Barki, respectively.

A fragment of 463 bp corresponding to Gaddi goat β-casein gene was investigated. A total of three SNPs were observed when compared with a template sequence (Acc. No. AF409096). All SNPs are given in Table 3. The average density of polymorphisms in caprine β-casein gene was 1 SNP per 154 bp sequenced. These variations are found to be synonymous in nature as these variations do not result in their corresponding amino acids. Chessa et al. (2008) reported that at least 9 variants have been found in goat β-CN (CSN2); 6 of them were characterized at the DNA level (A, A1, C, and E), whereas the other 3 variants were described only at the protein level. The recently identified silent A1 allele is characterized by a C > T transition at the 180th nucleotide of the ninth exon.

CSN3 exon 4 sequences from Indian goats (gaddi) were analyzed for SNP identifications. Compared with *Capra hircus* CSN3 (Coll et al., 1993), nine polymorphic nucleotide positions (245 T > C, 247G > A, 274G > A, 284A > G, 309A > G 345A > N, 384G > A, 471G > N and 509A > G), were identified in Indian goats. Most of the polymorphic sites were homozygous, except at position 471 (G > N) which was heterozygous for genotype AG in 50% of the Gaddi goats and 50% homozygous for genotype AA in the rest. Out of these, SNPs at 245, 247, 274, and 471 were reported earlier (Caroli et al., 2001, Yahyaoui et al., 2001, Angiolillo et al., 2002, Jann et al., 2004, Prinzenberg et al., 2005) in domestic goats. The SNPs at 245 (T > N) were silent, causing no change in amino acid at codon 43 in Gaddi goats. The SNPs at other positions, however, at codon 44, 53, 77, and 119 amino acids showed change of Gln to Arg, Asn to Ser, Gln to Arg, and Val to Ile, respectively. The codon 119 showed a heterozygous condition; while at all others were homozygous changes. The frequency of this allele was 0.16 in the Jakhrana goat breed (Gupta et al., 2009). The high number of similarity in polymorphic sites among most of the populations in different parts of the world is suggestive of similarity in evolutionary processes undergone by these populations (Kiplagat et al., 2010) (Table 4).

Comparative sequence analysis of the fragment covering partial exon 4 of CSN3 gene in the malpura breed of domestic sheep was identified with three nucleotide variations at the position 308 C > T, 630 G > A and 650 C > T and all these SNPs transition in nature and all are synonymous in nature, these SNPs will not effect CSN3 protein. Bastos et al. (2001) found a monomorphism at ovine CSN3 exon 4 by SSCP. Recently, an SNP was found by Light Cycler-based real-time PCR (Feligini et al., 2005) that did not result in an AA exchange and which showed a rather high frequency (22% and 12%) in the 2 breeds analyzed (Sarda and Paska). The low frequency of the T mutation at CSN3 makes this SNP less interesting, although an intriguing question about this locus is the high genetic variation found in cattle and goats (Prinzenberg et al., 1999, Yahyaoui et al., 2001, Yahyaoui et al., 2003, Jann et al., 2004) compared with the almost complete absence of variation in sheep deducible from literature, except for the synonymous mutation above mentioned (Feligini et al., 2005). When the malpura sheep were compared with goats, seven nucleotide changes were identified in sheep (Table 5).

The exon 7 of β-casein and exon 4 of κ-casein genes sequence of different breeds of livestock breeds were further subjected to basic local alignment search to know the sequence homology with the corresponding region of other species. BLAST analysis revealed homology of 99% with *Bos taurus* and *Bos indicus*, 97% with *Bubalus bubaline*, *99*% with *Bos grunniens*, 95% with *Ovis aries*, 95% with *Capra hircus* and 81% with *Camelus dromerius*.

## Conclusions

In conclusion, we demonstrate genetic variability in the β-casein and κ-casein genes of different livestock breeds. Although none of these SNPs caused an amino acid change, they could be in linkage disequilibrium with other functional allelic variants. These results will be useful in future works aimed at identifying possible associations with milk traits. This will help in determining if these polymorphisms are potential markers for milk production in Sahiwal cattle, Arunachali yak, malpura sheep and Gaddi goat for subsequent inclusion in selection programs.

## Figures and Tables

**Table 1:** Exon 7 of β-casein gene variants in domesticated Indian Sahiwal cattle and Arunachali yak.

Nucleotide position	*Bos taurus* (M55158)	Sahiwal cattle (*Bos indicus*) (KF430646)	Arunachali yak (HQ902899)	Type of change
8093	A	G	G	Transition
8101	A	C	C	Transversion
8261	T	T/C	C	Transition
8412	C	C	T	Transition
8491	T	C/T	T	Transition

**Table 2:** Exon 7 of β-casein gene variants in Gaddi goat.

Nucleotide position	Wild *Capra hircus* (AF409096)	Indian Gaddi goat	Type of change
11581	A	G	Transversion
11714	T	C	Transition
11790	T	C	Transition

**Table 3:** Exon 7 of β-casein gene variants in malpura sheep.

Nucleotide position	Wild *Ovis aries* (*X79703*)	Malpura sheep	Type of change
11803	G	A	Transversion
11936	C	T	Transition
12012	C	T	Transition

**Table 4:** κ-casein gene variants in domesticated Indian malpura sheep.

Nucleotide position	Exotic sheep (AY237637)	Indian sheep	Indian goat	Exotic goat (X60763)	Type of change
308	C	T	T	T	Transition
332	C	C	T	T	Transition
389	G	G	A	A	Transition
446	G	G	A	A	Transition
483	G	G	A	A	Transition
486	A	A	C	C	Transition
558	A	A	N	G	Transition
624	G	G	A	A	Transition
630	G	A	A	A	Transition
650	C	T	C	T	Transition

**Table 5:** κ-casein gene variants in domesticated Indian Gaddi goat.

Nucleotide position										
GenBank Acc. No.	245	247	274	284	309	345	384	471	509	Reference
X60763	T	A	A	G	G	A	G	G	A	Coll et al. (1993)
AF485340	T	A	A	G	G	A	G	A	A	Prinzenberg et al. (2005)
AF485341	C	A	A	A	A	A	G	A	G	Yahyaoui et al. (2001)
AY027868	C	G	A	G	A	A	G	A	A	Angiolillo et al. (2002)
AY090466	C	A	A	G	G	A	G	A	A	Yahyaoui et al. (2001)
AY166710	T	A	A	G	A	A	G	A	A	Jann et al. (2004)
AY350425	C	A	A	A	A	A	G	A	A	Prinzenberg et al. (2005)
AY428577	T	A	A	G	G	A	A	A	A	Prinzenberg et al. (2005)
D143734	T	A	A	G	G	A	G	G	A	
EF053350	T	A	A	G	G	A	G	N	A	Gupta et al. (2009)
EF053351	N	G	G	G	G	N	G	A	A	Gupta et al. (2009)
EF053356	T	G	G	G	G	A	G	G	A	Gupta et al. (2009)
EU584385	T	A	A	G	G	A	G	A	A	
EU584386	T	A	A	G	G	A	G	A	A	
This paper	T	A	A	G	G	A	G	N	A	
This paper	T	A	A	G	G	A	G	G	A	
This paper	T	A	A	G	G	A	G	N	A	
This paper	T	A	A	G	G	A	G	A	A	

## References

[bb0005] Angiolillo A., Yahyaoui M.H., Sanchez A., Pilla F., Folch J.M. (2002). Characterization of a new genetic variant in the caprine κ-casein gene. J. Dairy Sci..

[bb9000] Aschaffenburg R., Drewry J. (1955). Occurrence of different b-lactoglobulin in cow’s milk. Nature..

[bb0015] Bastos E., Cravador A., Azevedo J., Guedes-Pinto H. (2001). Single strand conformation polymorphism SSCP detection in six genes in Portuguese indigenous sheep breed “Churra da Terra Quente.”. Biotechnol. Agron. Soc. Environ..

[bb0010] Bonifacio C., Santos I.C., Belo C., Cravador A. (2001). SSCP analysis of alpha S1 casein, b-casein and k-casein genes in Charnequeira Portuguese indigenous goat breed. Arch. Zootech..

[bb0020] Caroli A., Jann O., Budelli E., Bolla P., Jager S., Erhardt G. (2001). Genetic polymorphism of goat κ-casein CSN3 in different breeds and characterization at DNA level. Anim. Genet..

[bb0025] Caroli A.M., Chessa S., Erhardt G.J. (2009). Milk protein genetic variation in cattle: impact on animal breeding and human nutrition. J. Dairy Sci..

[bb0030] Ceriotti G., Chessa S., Bolla P., Budelli E., Bianchi L., Duranti E., Caroli A. (2004). Single nucleotide polymorphisms in the ovine casein genes detected by polymerase chain reaction-single strand conformation polymorphism. J. Dairy Sci..

[bb9005] Chessa S., Budelli E., Gutscher K., Caroli A., Erhard G. (2003). Short communication: Simultaneous identification of five kappa-casein CSN3 alleles in domesticated goat by polymerase chain reaction single strand conformation polymorphism PCR-SSCP. J. Dairy Sci..

[bb0035] Chessa S., Rignanese D., Chiatti F., Radeghieri A., Gigliotti C., Caroli A. (2008). Technical note: simultaneous identification of CSN1S2 A, B, C, and E alleles in goats by polymerase chain reaction-single strand conformation polymorphism. J. Dairy Sci..

[bb9010] Coll A., Folch J.M., Sanchez A. (1993). Nucleotide sequence of the goat kappa-casein cDNA. J. Anim. Sci..

[bb0045] Feligini M., Valco S., Curik V.C., Parma P., Greppi G., Enne G. (2005). A single nucleotide polymorphism in the sheep kappa-casein coding region. J. Dairy Res..

[bb0040] Ferretti L., Leone P., Sgaramella V. (1990). Long range restriction analysis of the bovine casein genes. Nucleic Acids Res..

[bb0050] Grosclaude F., Mahe M.F., Brignon G., Di-Stasio L., Jeunet R.A. (1987). Mendelian polymorphism underlying quantitative variations of goat aS1-casein. Genet. Sel. Evol..

[bb0055] Gupta S.C., Kumar D., Pandey A., Malik G., Gupta N. (2009). New κ-casein alleles in Jakhrana goat affecting milk processing properties. Food Biotechnol..

[bb0060] Hayes H., Petit E., Bouniol C., Popescu P. (1993). Localization and as2–343 casein gene (CASAS2) to the homologous cattle, sheep and goat chromosomes 4 by in-situ hybridization. Cytogenet. Cell Genet..

[bb0065] He X.P., Xu X.W., Liu B. (2009). Molecular characterization, chromosomal localization and association analysis with back fat thickness of porcine LPIN2 and LPIN3. Mol. Biol. Rep..

[bb0075] Ikonen T., Ahlfors K., Kempe R., Ojala M., Ruottinen O. (1999). Genetic parameters for the milk coagulation properties and prevalence of noncoagulating milk in Finnish dairy cows. J. Dairy Sci..

[bb0070] Ikonen T., Bovenhuis H., Ojala M., Ruottinen O., Georges M. (2001). Associations between casein haplotypes and first lactation milk production traits in Finnish Ayrshire cows. J. Dairy Sci..

[bb0080] Jann O.C., Prinzenberg E.M., Luikart G., Caroli A., Erhardt G. (2004). High polymorphism in the kappa-casein CSN3 gene from wild and domestic caprine species revealed by DNA sequencing. J. Dairy Res..

[bb0085] Kiplagat S.K., Agaba M., Kosgey I.S., Okeyo M., Indetie D., Hanotte O., Limo M.K. (2010). Genetic polymorphism of kappa-casein gene in indigenous Eastern Africa goat populations. Int. J. Genet. Mol. Biol..

[bb0090] Lai X.S., Lan X.Y., Chen H., Wang X.L., Wang K.Y., Wang M., Yu H., Zhao M. (2009). A novel SNP of the Hesx1 gene in bovine and its associations with average daily gain. Mol. Biol. Rep..

[bb0095] Marletta D., Criscione A., Bordonaro S., Guastella A.M., D’Urso G. (2007). Casein polymorphism in goat’s milk. Lait.

[bb0100] Martin P., Szymanowska M., Zwierzchowski L., Leroux C. (2002). The impact of genetic polymorphisms on the protein composition of ruminants milks. Reprod. Nutr. Dev..

[bb0105] Moioli B., Pilla F., Tripaldi C. (1998). Detection of milk protein genetic polymorphism in order to improve dairy traits in sheep and goats: a review. Small Rumin. Res..

[bb0110] Nilsen H., Olsen H.G., Hayes B., Sehested E., Svendsen M., Nome T., Meuwissen T., Lien S. (2009). Casein haplotypes and their association with milk production traits in Norwegian Red cattle. Genet. Sel. Evol..

[bb0115] Oleński K., Cieslinska A., Suchocki T., Szyda J., Kaminski S. (2012). Polymorphism in coding and regulatory sequences of beta-casein gene is associated with milk production traits in Holstein-Friesian cattle. Anim. Sci. Paper Rep..

[bb0120] Othman O.E., El-Fiky S.A., Hassan N.A., Mahfouz E.R., Balabel E.A. (2013). Genetic variations of β- and K-casein genes in Egyptian sheep breeds. J. Appl. Biosci..

[bb0125] Popescu P., Long S., Riggs P., Womack J., Schmutz S., Fries R., Gallagher D.S. (1996). Standardization of cattle karyotype nomenclature: report of committee for standardization of the cattle karyotype. Cytogenet. Cell Genet..

[bb0130] Prinzenberg E.M., Krause I., Erhardt G. (1999). SSCP analysis at the bovine CSN3 locus discriminates six alleles corresponding to known protein variants A, B, C, E, F, G and three new DNA polymorphisms (H, I, AI). Anim. Biotechnol..

[bb9015] Prinzenberg E.M., Gutscher K., Chessa S., Caroli A., Erhard G. (2005). Caprine kappa-casein CSN3 polymorphism: new developments in molecular knowledge. J. Dairy Sci..

[bb0135] Rando A., Ramunno L., Masina P. (2000). Mutations in casein genes. Zootech. Nutr. Anim..

[bb0140] Sambrook J., Frotsch E.F., Maniatis T. (1989). Molecular Cloning: A Laboratory Manual.

[bb0150] Singh L.V., Rekha Sharma, Pandey A.K., Maitra A., Dixit S.P., Tripathi V., Mishra B.P. (2011). Identification of four novel single nucleotide polymorphism of CAPN1 gene in Indian goats. Indian J. Anim. Sci..

[bb0145] Singh Lakshya Veer, Sharma Anurodh, Kumari Namita, Kaur Navneet, Jayakumar S., Dixit S.P., Gupta Neelam, Gupta S.C. (2014). Comparative sequence analysis in the exon 5 of growth hormone gene in the various livestock species of India. Anim. Biotechnol..

[bb0155] Threadgill D.W., Womack J.E. (1990). Genomic analysis of the bovine milk protein genes. Nucleic Acids Res..

[bb0160] Velmala R., Vilkki J., Elo K., Mäki-Tanila A. (1995). Casein haplotypes and their association with milk production traits in the Finnish Ayrshire cattle. Anim. Genet..

[bb0165] Vinesh P.V., Brahma Biswajit, Kaur Rupinder, Datta Tirtha Kumar, Goswami Surender Lal, De Sachinandan (2013). Characterization of β-casein gene in Indian riverine buffalo. Gene.

[bb0170] Yahyaoui M.H., Coll A., Sanchez A., Folch J.M. (2001). Genetic polymorphism of the caprine kappa casein gene. J. Dairy Res..

[bb9020] Yahyaoui M.H., Angiolillo A., Pilla F., Sanchez A., Folch J.M. (2003). Characterization and genotyping of the caprine kappa casein variants. J. Dairy Sci..

